# Using Polygraph to Detect Passengers Carrying Illegal Items

**DOI:** 10.3389/fpsyg.2019.00322

**Published:** 2019-02-25

**Authors:** Runxin Yu, Si Jia Wu, Audrey Huang, Nathan Gold, Huaxiong Huang, Genyue Fu, Kang Lee

**Affiliations:** ^1^Department of Psychology, Hangzhou Normal University, Hangzhou, China; ^2^Department of Psychology, Zhejiang Normal University, Jinhua, China; ^3^Department of Applied Psychology and Human Development, University of Toronto, Toronto, ON, Canada; ^4^Department of Mathematics and Statistics, York University, Toronto, ON, Canada; ^5^The Fields Institute for Research in Mathematical Sciences, Toronto, ON, Canada

**Keywords:** lie detection, polygraph, comparison questions technique, electrocardiogram, galvanic skin response

## Abstract

The present study examined the effectiveness of a Modified-Comparison Questions Technique, used in conjunction with the polygraph, to differentiate between common travelers, drug traffickers, and terrorists at transportation hubs. Two experiments were conducted using a mock crime paradigm. In Experiment 1, we randomly assigned 78 participants to either a drug condition, where they packed and lied about illicit drugs in their luggage, or a control condition, where they did not pack or lie about any illegal items. In Experiment 2, we randomly assigned 164 participants to one of the two conditions in Experiment 1 or an additional bomb condition, where they packed and lied about a bomb in their luggage. For both experiments, we assessed participants’ RR interval, heart rate, peak-to-peak amplitude of Galvanic Skin Response (GSR) and all three combined, using Discriminant Analyses to determine the classification accuracy of participants in each condition. In both experiments, we found decelerated heart rates and increased peak-to-peak amplitude of GSR in guilty participants when lying in response to questions regarding their crime. We also found accurate classifications of participants, in both Experiment 1 (drug vs. control: 84.2% vs. 82.5%) and Experiment 2 (drug vs. control: 82:1% vs. 95.1%; bomb vs. control: 93.2% vs. 95.1%; drug vs. bomb: 92.3% vs. 90.9%), above chance level. These findings indicate that Modified-CQT, combined with a polygraph test, is a viable method for investigating suspects of drug trafficking and terrorism at transportation hubs such as train stations and airports.

## Introduction

Millions of people travel all over the world everyday, whether for work, vacation, or family and friends. In the midst of masses transporting from one location to the next, such transportation hubs as airports and train stations have become a prime setting for criminal activities such as smuggling and terrorism. Thus, there is great need for transportation authorities to accurately identify individuals carrying illegal items in their luggage. However, few studies have examined potential methods to assist transportation authorities in detecting whether a passenger is carrying illegal items. To bridge this gap, the present research investigated the effectiveness of using polygraph along with a Modified Comparison Questions Technique (CQT) to detect passengers lying about carrying illegal items in their luggage.

Throughout history, various methods have been used to detect lies. Studies show that deception has largely been observed from facial expressions (Su and Levine, 2016), neural activities (Hu X.S. et al., 2012; Farah et al., 2014; Bhutta et al., 2015; Hong and Santosa, 2016; Hong and Khan, 2017; Hong et al., 2017, 2018), and changes in the Autonomic Nervous System (ANS; Verschuere et al., 2004). The motivation behind such investigations into deception is largely to identify liars, thieves, and criminals for the safety and security of society. Thus, many researchers have designed experiments with a “mock crime” (Ben-Shakhar, 2002), simulating offenses that may occur in real life. A component of the “mock crime” involves raising the stakes of the situation such that the participant may experience emotions and stress associated with a guilty act and when they later lie about the act. For example, researchers may give participants the opportunity to choose whether or not to steal a check written out to a group with which they politically disagree (Tsiamyrtzis et al., 2007). Such an experimental design not only allows participants the choice to “commit a crime”, it also triggers emotional reactions to the scenario itself as the act becomes something they care about (e.g., hindering their opposing political group). Thus, when “guilty” participants are questioned about the act, they are more likely to react in an emotional and realistic way, allowing researchers to observe facial, neural, and autonomic reactions representative of real criminals.

While extensive research has investigated lying in the context of crimes already committed (e.g., theft), few have explored lying with the intention of committing a crime (e.g., drug smuggling or terrorism). Specifically, security at transportation hubs are expected to successfully identify those with smuggling drugs or committing terrorism when the criminal act is being committed. However, existing research have yet to identify an effective method to detect crimes in such situation (Weinberger, 2010). Our present study aims to identify passengers carrying illegal items (e.g., drugs or bomb) within a mock airport security scenario.

In order to detect passengers carrying illegal items, we observed autonomic reactions during a mock security screening, specifically by analyzing results obtained by a polygraph. The polygraph (Reid and Inbau, 1977) is a device that indirectly assesses psychological processes, such as lying, through analyses of changes in the ANS (Raskin, 1979, 1986; Lykken, 1998). To be precise, the polygraph uses such physiological measurements such as ElectroCardioGraphy (ECG) and Galvanic Skin Response (GSR) to determine pulse and skin conductivity changes that correspond with deception. The polygraph was chosen as our lie detection device given consideration that facial expressions may be unreliable indicators of lies. It is well established that law enforcement’s common use of such non-verbal cues as facial expression has yielded poor deception detection results (Vrij, 2004; Weinberger, 2010).

In the present study, the application of the polygraph was paired with a novel questioning paradigm we constructed for detection of passengers carrying illegal items. Several questioning paradigms have been used extensively in conjunction with the polygraph for lie detection. Each paradigm involves a set of questions to ask suspects with the purpose of better extracting physiological responses that accurately represent psychological states relevant to the crime. The Relevant/Irrelevant (R/I; Keeler, 1930) paradigm was the first of these to be developed, asking suspects relevant questions (i.e., those regarding the crime) and irrelevant questions (i.e., those regarding personal information). The Comparison Question Technique (CQT; Reid, 1947) built on the R/I paradigm by adding a third type of questions: comparison questions regarding moral character (Synnott et al., 2015). The comparison questions were meant to help differentiate guilty suspects from innocent suspects whose physiological responses indicated high stress toward relevant questions (Meijer and Verschuere, 2015). This technique was most widely used by law enforcement agencies (Reid, 1947; Raskin and Honts, 2002; Meijer and van Koppen, 2008). However, most researchers find it lacking in scientific foundation (e.g., Lykken, 1974; Ben-Shakhar, 2002; Iacono and Lykken, 2002; National Research Council, 2003). Nevertheless, this technique is widely used and favored by many investigators worldwide due to the fact that it does rely on the key assumptions underlying the technique favored by scientists in the laboratories to be discussed below.

In contrast to the CQT, the Concealed Information Technique (CIT; Verschuere et al., 2011), otherwise known as the Guilty Knowledge Technique (GKT; Lykken, 1959), is acclaimed for its scientific basis. The CIT is based on the Orienting Response (OR; Sokolov, 1963; Lynn, 1966), involving changes to heart rate and skin conductance to a significant stimulus. Utilizing relevant questions, the CIT observes for OR by presenting suspects with answers to the questions, including the correct answer. It is expected that the guilty person would react strongly when presented with information that is significant to the crime whereas an innocent person would have no change in reaction between the correct answer and the insignificant alternatives (Lykken, 1974). In addition, the administrators of the CIT must be unaware of the details of the crime to ensure that they do not influence the results, adding to the validity of the paradigm (Meijer et al., 2016).

Despite the scientific validity of the CIT, it is rarely used by law enforcements around the world; Japan is perhaps the only country that uses the CIT during investigations (Yamamura and Miyata, 1990; Hira and Furumitsu, 2002; Nakayama, 2002; Osugi, 2011). The reason behind such limited application of the CIT may be due to difficulties in formulating effective questions (Meijer and Verschuere, 2015). Further, the validity of the CIT relies on two assumptions: (1) criminals will remember details of their crime perfectly, and (2) only the criminal would know details unique to the crime. Such may not always be the case as crimes of passion (i.e., those committed under impulse) and crimes committed by those suffering from mental illness (e.g., schizophrenia) may not remember certain details of the crime; under such conditions, the CIT fails to produce Orienting Responses (Carmel et al., 2003; Gamer et al., 2010; Nahari and Ben-Shakhar, 2011; Peth et al., 2012). Moreover, it can be difficult to ensure that details of the crime are only known by the criminal because innocent witnesses may share the same knowledge and so too would many others if media coverage of on-going investigations have revealed the same details.

Given the advantages and disadvantages of both CQT and CIT, the present research has formulated an improved paradigm, called Modified Comparison Questions Technique (Modified-CQT). Modified-CQT combines the structure of the traditional CQT with the scientific method of the CIT. Specifically, the Modified-CQT consist of questions regarding basic personal information and common travel items as questions to allow comparison of physiological states during truth and lie-telling. In addition, relevant questions regarding crimes are included to assess for OR.

To test the validity of the Modified-CQT, we set up a “mock crime” (Ben-Shakhar, 2002) in which to evaluate the effectiveness of Modified-CQT, specifically in the context of passengers carrying illegal items in their luggage. We conducted two experiments, both set at a laboratory simulated transportation security scene. We set up the scene by asking participants to pack a number of common travel items, with some asked to pack an additional illegal item, into a carry-on suitcase. Then we informed participants that they will be traveling with the suitcase and prepared them to answer questions that they will likely encounter with a customs officer. Those who were asked to pack an illegal item, were instructed to lie about their possession of the item. Next, participants were led to the testing room where another experimenter who was unaware of whether they were carrying an illegal item in their luggage asked them a series of questions. We measured the participants’ physiological responses based on ECG and GSR signals. To show participants the effectiveness of the polygraph test, we conducted a rigged card test (see section “Method” in Experiment 1) before proceeding to question them.

The conditions of the two experiments are where they differ. In Experiment 1, the guilty condition involved the task of packing and carrying fake drugs in the carry-on suitcase while the control condition (i.e., innocent condition) involved simply carrying common items, nothing illegal. We assessed the effectiveness of the Modified-CQT in detecting participants who were carrying an illegal item in their luggage versus those who were not. In Experiment 2, we added another guilty condition which involved the task of packing and carrying a bomb; thus participants were randomly assigned to one of three conditions: to either carry fake drugs, a fake bomb, or no illegal items. This experiment assessed the effectiveness of the Modified-CQT to differentiate not only participants carrying illegal items versus innocent participants, but also between participants carrying a fake bomb vs. fake drugs.

## Experiment 1

### Method

#### Participants

Eighty-three undergraduate students between 18 and 24 years of age from Zhejiang Normal University participated in the study. Among them, five participants were excluded due to failure to comply with the protocol (e.g., participant lied in response to questions regarding basic personal information). The final sample consisted of seventy-eight participants (10 males; mean age = 20.24; *SD* = 1.24), 40 of which were in the control condition and 38 in the drug condition. All participants read and signed a consent form prior to the experiment. The present study was approved by the Research Ethics Review Committee at Zhejiang Normal University.

#### Materials

Five types of common travel items (clothes, toiletries, sandals, books, and sunglasses) and 1 illegal item (fake drugs) were provided to participants on a table in the staging room, where we set up the mock crime. Participants were also given a carry-on suitcase in which to pack the items. Common travel items were chosen to be compatible with the gender of the participant, thus 2 sets of travel items were kept on hand. Only participants who were randomly assigned to the guilty condition were given the 1 illegal item to pack. A blank sheet of paper was given to participants for them to recall the items they had packed into the suitcase.

A deck of six poker cards was used for the rigged card test. The deck of cards was always presented, face down, in the same sequence (3 of Clubs, 7 of Clubs, 10 of Diamonds, 8 of Diamonds, 6 of Spades, and 5 of Hearts) to ensure that the experimenter would know which card the participant later picked.

The FDA approved BIOPAC physiological measurement system, BIOPAC MP150 (BIOPAC Systems, Inc., Goleta, CA, United States) was used to collect ECG and GSR data. Specifically, the electrocardiogram amplifier module (ECG100C) and the electrodermal activity amplifier module (GSR100C) were connected to the BIOPAC system to record ECG and GSR signals, respectively. A three-lead configuration was used with the ECG100C module: The White lead was connected to SHIELD and VIN- on the module, the Red lead was connected to SHIELD and VIN+, and the Black lead was connected to GND. Disposable electrodes were placed on participants’ skin to obtain ECG data. A set of two Ag-AgCl electrodes (TSD203) was connected to the GSR100C module. The electrodes had stretchable Velcro straps and a hole at the center of each, which was filled with GEL101 to allow contact with the skin and measure of GSR signals. Alcohol swabs were used to clean participants’ skin prior to the attachment of electrodes. The BIOPAC MP150 system was set at 0.5–35 Hz band for the ECG signal and at the 0.05–1 Hz band for the GSR signal. ECG and GSR signals were displayed on a laptop, sampled at 1000 Hz, using AcqKnowledge v. 4.1 (BIOPAC Systems, Inc., Goleta, CA, United States).

The Modified-CQT was formulated with 21 Yes/No questions (Appendix 1), including 10 questions regarding personal information and 11 relevant questions regarding items in the carry-on suitcase. In order to construct the 10 questions regarding personal information in the Modified-CQT, a questionnaire (Appendix 2) was used to obtain some basic personal information from each participant prior to the mock crime. The 11 relevant questions were formed based on the possession/non-possession of items in the suitcase. The questions were presented to participants on a computer screen, using E-prime 2.0. The computer screen was placed on a table in the center of the testing room.

#### Procedure

**FIGURE 1:**
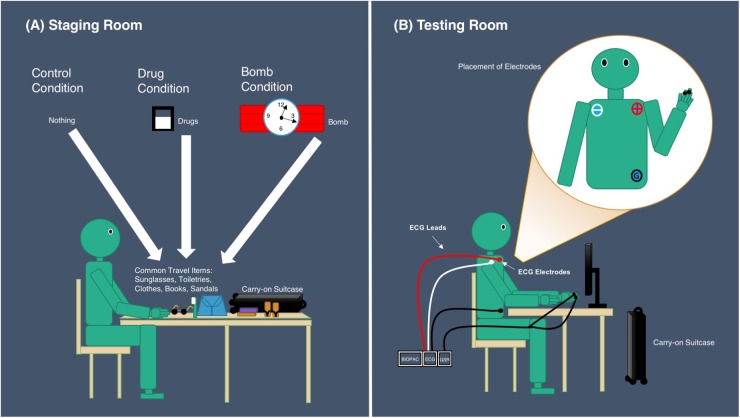
**(A)** Set up of staging room. Note that the addition of the bomb condition only occurs in Experiment 2. **(B)** Set up of testing room.

Participants were tested individually under the supervision of two experimenters. Experimenter 1 was responsible for setting up the mock crime in the staging room, whereas Experimenter 2 was responsible for conducting the interrogation to establish the participant’s guilt or innocence in the testing room (see Figure 1). Experimenter 2 was blind to the condition to which the participants were assigned. This was achieved as follows:

Prior to the participant’s arrival, Experimenter 1 randomly assigned the participant into one of two conditions, the drug condition or the control condition. Next, Experimenter 1 prepared travel items, choosing the set that is compatible with the participant’s gender, which was affirmed when scheduling the research session. If the participant was assigned into the drug condition, Experimenter 1 also prepared fake drugs as an item to pack. Upon arrival, the participant was given a verbal and written overview of the study and its tasks, a consent form to sign, and a questionnaire (Appendix 2) to complete regarding his/her personal information. The completed questionnaire was given to Experimenter 2 to formulate questions in the Modified-CQT regarding personal information. Next, Experimenter 1 led the participant into a room with items previously prepared and a carry-on suitcase on a table. Experimenter 1 then instructed the participant to pack the items on the table into the suitcase. Once this is done, the participant is asked to recall the items they had packed by writing the items on a blank sheet of paper. If the participant did not recall the items correctly, they will be asked to repack the suitcase and try recall again until correct recall is achieved. If the participant correctly recalled all items they packed into the suitcase, Experimenter 1 moved on to instruct the participant on answering questions regarding items of the suitcase. Specifically, participants were instructed to answer each question truthfully and if participants was assigned to the drug condition, they were instructed to answer all questions truthfully, except for questions regarding possession of drugs. Finally, Experimenter 1 led participants with the suitcase to the testing room where they were then interrogated on the contents of the suitcase, and then left the scene.

In the testing room, Experimenter 2 greeted participants by informing them that they were suspected of carrying illegal items in the suitcase and a polygraph test must be conducted to determine their guilt or innocence. Next, Experimenter 2 placed ECG electrodes, onto participants, based on Einthoven’s triangle, with an ECG lead attached to each electrode: near the right shoulder (White lead), left shoulder (Red lead), and left hip (Black lead). Then, the TSD203 electrodes were placed on participants’ left index and middle fingers. Following this, Experimenter 2 administered the rigged card test, getting participants to pick a card from the deck of 6 cards, faced down, and view it without showing the experimenter. Experimenter 2 questioned participants on which card they had picked, instructing the participant to respond “no” to all questions. Having known the sequence of the cards and thus the card participants had picked, Experimenter 2 informed participants that the polygraph was able to determine which response of “no” was a lie and therefore the card that they had picked is known. The rigged card test was a common practice in the field polygraph testing. It was conducted prior to administering the Modified-CQT to illustrate participants of the “validity” of the polygraph in the hope to increase “guilty” participants’ anxiety for later questioning (Lewis and Cuppari, 2009).

**FIGURE 2:**
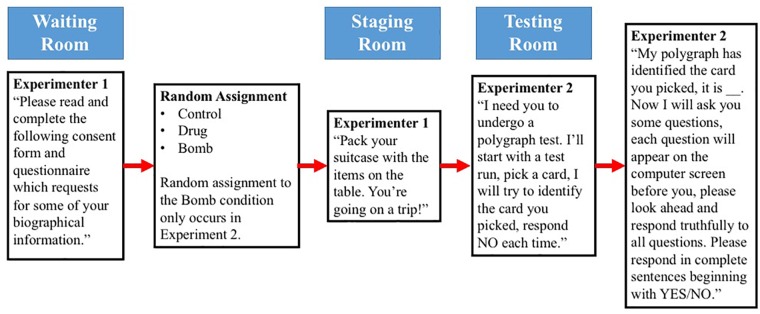
Layout of experimental procedure from perspective of participant.

Next, Experimenter 2 administered the Modified-CQT with the polygraph. Participants were asked to sit before a computer screen where questions of the Modified-CQT were presented in the form of text, upon appearance of which, Experimenter 2 would ask the question verbally, proceeding to the response time when he finished asking the question. Participants were asked to respond truthfully to all questions with “Yes” or “No,” followed by a complete statement on the subject of the questions. For example, if asked “Are you male?”, participants were asked to respond, “Yes, I am male” or “No, I am not male.” Presentation of each question varied slightly depending on the amount of time it took for Experimenter 2 to finish asking the questions, usually 3–5 s. The participant was first presented with questions regarding personal information, then questions regarding contents of the suitcase. Each question was randomly displayed 4 times within its own group of questions. In addition, the beginning and end of each question was marked by a “beep” sound, with interval of 10 s between questions for participants to respond to questions. The “beep” sound was programmed into E-prime, such that activation of the sound would also trigger a marker to be made onto the data being recorded on AcqKnowledge, synchronizing the ECG and GSR responses to each question. Figure 2 shows the experimental procedure from the perspective of the participant.

#### Data Analysis

##### Feature extraction

**FIGURE 3:**
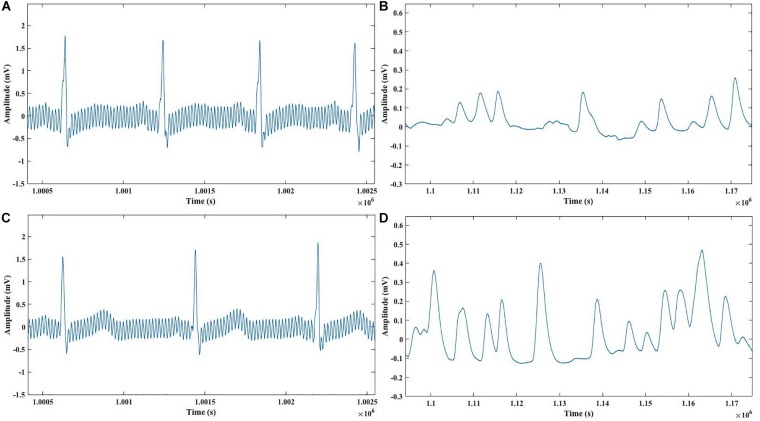
**(A)** Raw ECG signals of a participant in the Control condition during questioning period. **(B)** Raw GSR signals of a participant in the Control condition during questioning period. **(C)** Raw ECG signals of a participant in the Drug condition during questioning period. **(D)** Raw GSR signals of a participant in the Drug condition during questioning period.

Each participant’s raw ECG and GSR data was processed using MATLAB (The MathWorks, Inc.) to extract features from ECG and GSR signals to detect significant physiological changes. Figure 3 illustrates an example of raw ECG and GSR signals from both control and drug condition participants.

**FIGURE 4:**
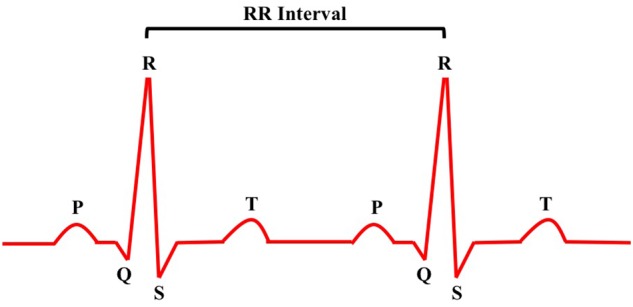
RR interval.

For the ECG data, we calculated the RR intervals (RRI; Figure 4), which is the time interval between successive R-waves. As shown in Figure 4, in a typical ECG signal, there are several key characteristic waves such as the P, Q, R, S, T waves. According to the extensive literature, the interval between two successive R wave peaks (RR interval or RRI) is typically used to obtain heart rate variability features and to calculate heart rate. For this reason, we obtained, from our ECG signals, both RRI and heart rate relating to each question.

For the GSR data, we calculated the peak-to-peak amplitude (the change between peak and trough amplitude value), of GSR wave within the question and response time. Thus, RRI, heart rate, and GSR amplitude were selected as physiological features to be analyzed for indication of lies because these features are one of the most commonly evaluated in lie detection research (Raskin, 1979, 1986; Lykken, 1998). Having calculated these features, we categorized the questions into three types: basic personal information, carrying common travel items, and carrying drugs. All indices were converted to the *z*-score in order to correct for individual differences.

##### Classification

Participants were assigned to either the control condition, where they carried no illegal items or the drug condition, where they carried fake drugs. As such, those assigned to the control condition would have told the truth to all questions, being innocents, whereas those assigned to the drug condition would have told a lie to the question regarding whether they were carrying drugs, being “criminals.” Thus, the binary classification including control vs. drug was used to differentiate “criminals” from innocents.

To implement the binary classification, Discriminant Analyses (DA; Fisher, 1936) were conducted to analyze the accuracy of the identification of criminals and innocents. This was done by building a separate function with each one of the three physiological indices (RR interval, heart rate, peak-to-peak amplitude of GSR), as well as all three indices combined, across types of questions (personal information, travel items, drug). In keeping with previous research, parameter A′, based on Signal Detection Theory (SDT) proposed by Grier (1971), was adopted. The value of Grier’s A′ represents the area under the Receiver Operating Characteristic (ROC) curve ranging between the threshold 0.5–1 (Fogarty et al., 2005), corresponding to none and perfect discrimination, respectively (National Research Council, 2003; Hu X. et al., 2012). In the present study, Grier’s A′ measures the ability to discriminate between participants guilty of carrying drugs and those innocent of crime, carrying nothing illegal. It is defined as follows:

(1) $$A\prime =\frac{1}{2}+(Hits-False\;alarms)\frac{1+Hits-False\;alarms}{4(Hits)(1-False\;alarms)}$$

For Equation 1 and our report of results below, for the Drug condition, we defined Hits as instances where we correctly identified a participant who was carrying “drugs” as lying about possession of drugs. We defined False Alarms as instances where we incorrectly identified a participant who was not carrying “drugs” as lying about possession of drugs. For the Control condition, we defined Hits as instances where we correctly identified a participant who was not carrying illegal items as telling the truth about not carrying drugs. We defined False Alarms as instances where we incorrectly identified a participant who was carrying “drugs” as telling the truth about not carrying drugs.

#### Results and Discussion

##### Physiological indices

The *z*-scores corresponding to the RR interval, heart rate, and peak-to-peak amplitude of GSR were averaged across participants within the two conditions (control and drug) and across the three types of questions (basic personal information, carrying common travel items, and carrying drugs).

The 2 conditions × 3 types of questions repeated analyses of variance (ANOVAs) were conducted for each physiological index, respectively. The conditions served as two levels of between-subjects factor, and the types of questions served as three levels of within-subjects factor. The dependent variables were the mean *z*-scores of three physiological indices across types of questions within each condition.

**FIGURE 5:**
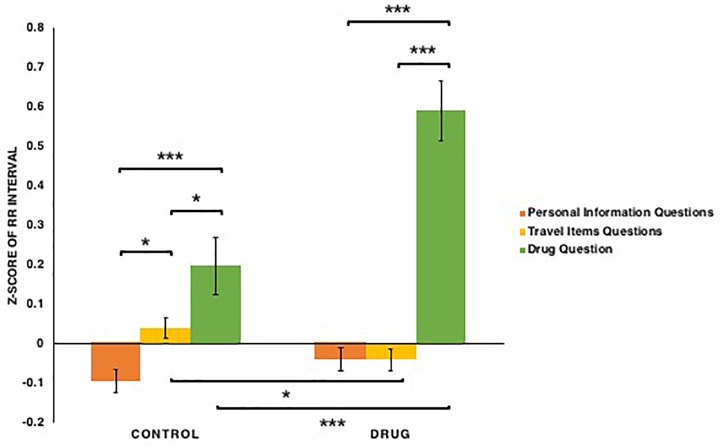
The *z*-scores of the RR interval among participants in the control and drug conditions when answering three types of questions: basic personal information, carrying common travel items, carrying drugs. Note that ^∗^*p* < 0.05, ^∗∗^*p* < 0.01, ^∗∗∗^*p* < 0.001. The error bars stand for standard errors (SEs).

For RR intervals (see Figure 5), both conditions [*F*(1,76) = 15.147, *p* < 0.001, ${\text{η}}_{\text{p}}^{2}$ = 0.166] and types of questions [*F*(2,152) = 43.227, *p* < 0.001, ${\text{η}}_{\text{p}}^{2}$ = 0.363] had significant main effects, as did the conditions × types of questions interaction: *F*(2,152) = 10.419, *p* < 0.001, ${\text{η}}_{\text{p}}^{2}$ = 0.121. This interaction appeared to reflect the fact that for the participants in both control [*t*s(39) > 2.00, *p*s < 0.05] and drug conditions [*t*s(37) > 7.04, *p*s < 0.001, *Bonferroni correction*], RR intervals when answering the drug question were shown to be longer than when answering questions regarding basic personal information and common travel items. Similarly, when answering the drug question, RR intervals of participants in the drug condition were shown to be longer than that of those in the control condition: *t*(76) = 3.72, *p* < 0.001, *Bonferroni correction*. In addition, for the participants in the control condition, RR intervals when answering questions regarding common travel items were shown to be longer than when answering basic personal information questions: *t*(39) = 2.44, *p* = 0.017. Thus, these results provide evidence that all participants experience increase in their RR intervals when answering the drug question, with a stronger increase for those in the drug condition, whereas those in the control condition, experience shorter RR intervals when they answered questions of basic personal information as compared to those of common travel items.

**FIGURE 6:**
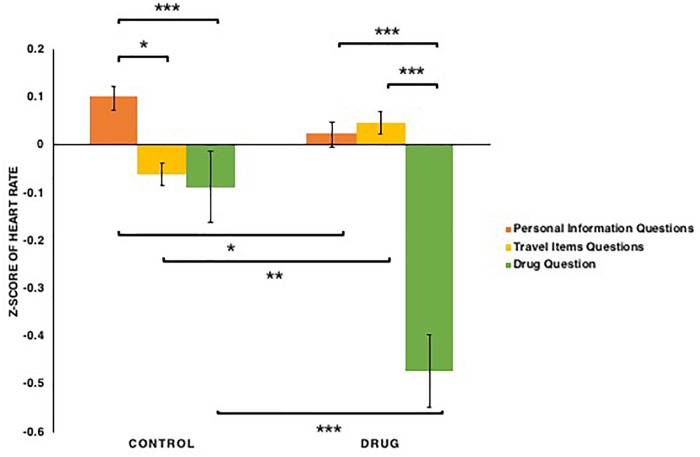
The *z*-scores of the heart rate among participants in the control and drug conditions when answering three types of questions: basic personal information, carrying common travel items, carrying drugs. Note that ^∗^*p* < 0.05, ^∗∗^*p* < 0.01, ^∗∗∗^*p* < 0.001. The error bars SEs.

For heart rate (see Figure 6), both conditions [*F*(1,76) = 14.08, *p* < 0.001, ${\text{η}}_{\text{p}}^{2}$ = 0.156] and types of questions [*F*(2,152) = 24.395, *p* < 0.001, ${\text{η}}_{\text{p}}^{2}$ = 0.243] had significant main effects, as did the conditions × types of questions interaction: *F*(2,152) = 11.595, *p* < 0.001, ${\text{η}}_{\text{p}}^{2}$ = 0.132. This interaction appeared to reflect the fact that for the participants in the drug condition, heart rates when answering the drug question were lower than when answering questions regarding basic personal information and common travel items [*ts*(39) < -5.66, *p*s < 0.001, *Bonferroni correction*]. For the participants in the control condition, heart rates when answering the drug and travel items questions were lower than when answering personal information questions only [*t*(39) = -2.18, *p* = 0.032]. Similarly, when answering the drug question, heart rates of participants in the drug condition was lower than that of those in the control condition: *t*(76) = -3.63, *p* < 0.001, *Bonferroni correction*. In addition, for the participants in the control condition, heart rates when answering questions regarding travel items was lower than when answering personal information questions: *t*(39) = -3.39, *p* = 0.047. Thus, these results provide evidence that all participants experience decrease in their heart rate when answering the drug question, with a stronger decrease for those in the drug condition, whereas those in the control condition experience higher heart rates when they answered personal information questions compared to the travel items questions.

**FIGURE 7:**
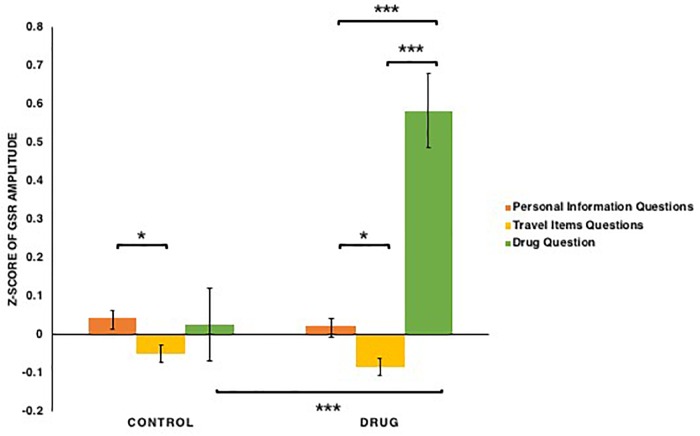
The *z*-scores of the peak-to-peak amplitude of GSR among participants in the control and drug conditions when answering three types of questions: basic personal information, carrying common travel items, carrying drugs. Note that ^∗^*p* < 0.05, ^∗∗^*p* < 0.01, ^∗∗∗^*p* < 0.001. The error bars SEs.

For peak-to-peak amplitude of GSR (see Figure 7), both conditions [*F*(1,76) = 17.355, *p* < 0.001, ${\text{η}}_{\text{p}}^{2}$ = 0.186] and types of questions [*F*(2,152) = 18.938, *p* < 0.001, ${\text{η}}_{\text{p}}^{2}$ = 0.199] had significant main effects, as did the conditions × types of questions interaction: *F*(2,152) = 14.473, *p* < 0.001, ${\text{η}}_{\text{p}}^{2}$ = 0.16. This interaction appeared to reflect the fact that for participants in the drug condition, galvanic skin response amplitudes when answering the drug question were shown to be greater than when answering questions regarding personal information and travel items: *t*s(39) > 5.25, *p*s < 0.001, *Bonferroni correction*, whereas in the control condition GSR amplitudes did not differ significantly when answering different types of questions. Furthermore, when answering the drug question, GSR amplitude of participants in the drug condition was shown to be greater than that of those in the control condition: *t*(76) = 4.10, *p* < 0.001, *Bonferroni correction*. In addition, for participants in both control [*t*(39) = 2.04, *p* = 0.045] and drug conditions [*t*(37) = 2.31, *p* = 0.023] GSR amplitudes when answering personal information questions were shown to be greater than when answering the travel items questions. Thus, these results provide evidence that in the drug condition, participants’ GSR amplitudes increase when they answered the drug question, and in both conditions, participants’ GSR amplitudes increase when they answered personal information questions compared to the travel items questions.

##### Group classifications

The Discriminant Analyses results revealed a significant difference between the control and drug conditions based on the canonical discriminant function: Wilk’s λs < 0.801, *p*s < 0.01, used in the analysis. The classification results (see Table 1), using all indices, revealed that participants could be best categorized as drug traffickers/common travelers (participants in the drug/control condition), with an overall accuracy of 83.4%. For drug traffickers, we achieved a Hit rate (i.e., guilty participants correctly classified as guilty) 84.2% and a False Alarm rate (i.e., guilty participants incorrectly classified as innocent) of 15.8%. For common travelers, we achieved a Hit rate (i.e., innocent participants correctly classified as innocent) of 82.5% and a False Alarm rate (i.e., innocent participants incorrectly classified as guilty) of 17.5%. The corresponding standardized canonical discriminant function coefficients can be seen in Table 2. The physiological indices that contributed most, with largest coefficients, to the classification of drug trafficker vs. common travelers were RR intervals and heart rates when answering personal information and travel items questions, as well as the RR intervals when answering the drug question.

**Table 1:** Hit rates (i.e., correct classification rates) of guilty and innocent participants, using one of three and all physiological indices in Experiment 1.

Condition 1 vs. Condition 2	Indices	Wilk’s λ	*p*	Hit rate of condition 1	Hit rate of condition 2
Drug condition	RR interval	0.769	0.001	73.7%	77.5%
vs.	Heart rate	0.737	<0.001	68.4%	80.0%
Control condition	GSR amplitude	0.801	0.003	55.3%	77.5%
	All	0.512	<0.001	84.2%	82.5%

**Table 2:** Standardized canonical discriminant function coefficients in Experiment 1.

	Function
Comparison between conditions	Drug condition vs. Control condition
RR Interval of Personal Information Questions	3.8
RR Interval of Travel Items Questions	3.972
RR Interval of Drug Question	1.196
Heart Rate of Personal Information Questions	1.197
Heart Rate of Travel Items Questions	1.991
Heart Rate of Drug Question	-0.048
GSR Amplitude of Personal Information Questions	-0.286
GSR Amplitude of Travel Items Questions	-0.53
GSR Amplitude of Drug Question	0.633

Thus, Experiment 1 revealed that when guilty participants (those in the drug condition) tried to conceal information about possession of an illegal item (i.e., lying in response to the drug question), their RR intervals increased, heart rates decelerated, and skin conductance increased. In addition, our Discriminant Analyses (DA) showed: 84.2% accuracy for identifying guilty participants and 82.5% accuracy for identifying innocent participants. These results clearly indicate our success at differentiating between participants carrying fake drugs from those without any illegal items, thus it is evident that our Modified-CQT, when used in conjunction with the polygraph test, is an affective paradigm for determining guilt versus innocence. One limitation of Experiment 1 is that it only includes one guilty condition. It is unclear whether our method could be used to differentiate individuals committing different crimes. Experiment 2 was conducted with more participants and with an added guilty condition of participants carrying a fake bomb, ultimately to determine if the Modified-CQT would not only successfully differentiate between innocence and guilt, but also different types of guilt.

## Experiment 2

### Methods

#### Participants

One hundred and sixty-nine undergraduate students between 17 and 27 years of age from Zhejiang Normal University participated in the study. Among them, five participants were excluded due to failure to comply with the protocol (e.g., participant lied in response to questions regarding basic personal information). The final sample consisted of one hundred and sixty-four participants (24 males; mean age = 20.27; *SD* = 1.5), including eighty participants in the control condition and 42 in the drug and bomb conditions, respectively. All participants read and signed a consent form prior to the experiment. The present study was approved by the Research Ethics Review Committee at Zhejiang Normal University.

#### Materials

Experiment 2 utilized the same materials as those in Experiment 1, only with the addition of the fake bomb provided in the staging room if the participant was randomly assigned to the added guilty condition where participants were asked to pack and carry a fake bomb (see Figure 1). In addition, the corresponding question regarding the possession of a bomb was included in the Modified-CQT for Experiment 2.

#### Procedure

Experiment 2 was conducted in the same manner as Experiment 1, with the exception for participants in the bomb condition, who were instructed to pack and carry a fake bomb in the suitcase and lie to the question regarding possession of a bomb.

#### Data Analysis

Data analysis for Experiment 2 was conducted in the same manner as Experiment 1, with the exception that given the addition of the bomb condition, we categorized questions into four types: basic personal information, carrying common travel items, carrying drugs, and carrying a bomb. We then analyzed for changes in RR interval, heart rate, and peak-to-peak amplitude of GSR in response to each type of questions for each condition.

Further, with the addition of the bomb condition, there was a need to implement more classifications. Thus, the binary classifications including control vs. drug, control vs. bomb were used to differentiate criminals from innocents. In addition, the binary classification of drug vs. bomb was used to differentiate between different types of criminals.

Grier’s A′ (Equation 1) was calculated same as before, with the addition of calculations for the Bomb condition, where we defined Hits as instances where we correctly identified a participant who was carrying a “bomb” as lying about possession of a bomb. We defined False Alarms as instances where we incorrectly identifies a participant who was not carrying a “bomb” as lying about possession of a bomb. For the Control condition, we defined Hits as instances where we correctly identified a participant who was not carrying illegal items as telling the truth about not carrying drugs and a bomb. We defined False Alarms as instances where we incorrectly identified a participant who was carrying “drugs” or a “bomb” as telling the truth about not carrying drugs or a bomb.

#### Results and Discussion

##### Physiological indices

The *z*-scores corresponding to the RR interval, heart rate and peak-to-peak amplitude of GSR, were averaged across participants within the three conditions (control, drug, and bomb) and across the four types of questions (basic personal information, carrying common travel items, carrying drugs, and carrying bomb).

The 3 conditions × 4 types of questions repeated ANOVAs were conducted for each physiological index, respectively. The condition served as three levels of between-subjects factor, and the types of questions served as four levels of within-subjects factor. The dependent variables were the mean *z*-scores of three physiological indices across types of questions within each condition.

**FIGURE 8:**
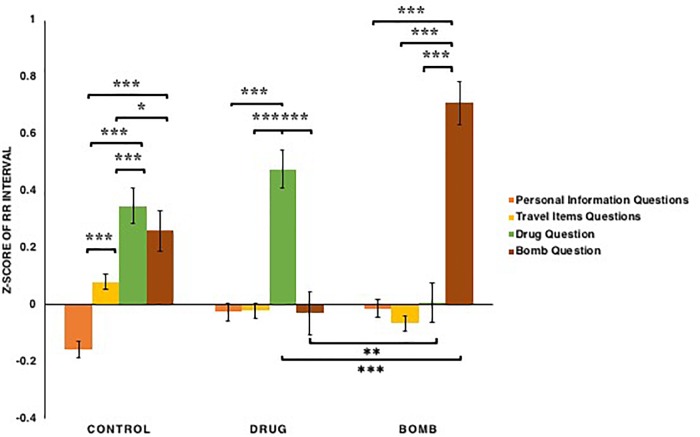
The *z*-scores of the RR interval among participants in the bomb, control and drug conditions across the four types of questions: basic personal information, carrying common travel items, carrying drugs, carrying a bomb. Note that ^∗^*p* < 0.05, ^∗∗^*p* < 0.01, ^∗∗∗^*p* < 0.001. The error bars SEs.

For RR intervals (see Figure 8), the types of questions had a significant main effect: *F*(3,363) = 36.937, *p* < 0.001, ${\text{η}}_{\text{p}}^{2}$ = 0.234, as did the conditions × types of questions interaction: *F*(6,363) = 23.185, *p* < 0.001, ${\text{η}}_{\text{p}}^{2}$ = 0.277. This interaction appeared to reflect the fact that for the participants in both drug [*t*s(38) > 5.74, *p*s < 0.001, *Bonferroni correction*] and bomb conditions [*t*s(43) > 8.11, *p*s < 0.001, *Bonferroni correction*], RR intervals when answering the questions that matched their crimes (e.g., participants in the drug condition when answering the drug question or those in the bomb condition when answering the bomb question) were detected to be longer than when answering other questions, whereas for participants in the control condition, RR intervals when answering the illegal items questions (the drug and bomb questions) were detected to be longer than when answering other questions (the personal information and travel items questions): *t*s(40) > 2.38, *p*s < 0.05. However, in terms of the RR intervals of participants in both drug and bomb conditions, when answering questions that mismatched their crimes (e.g., participants in the drug condition when answering the bomb question or those in the bomb condition when answering the drug question), were detected as shorter than in other conditions: *t*s(121) < -2.70, *p*s < 0.01. Additionally, for participants in the control condition, RR intervals when answering travel items questions were detected as longer than when answering the personal information questions: *t*(40) = 4.26, *p* < 0.001, *Bonferroni correction*. Thus, these results provide evidence all participants experience increase in their RR intervals when answering illegal items questions, especially those in drug and bomb conditions when answering questions that matched their crimes. In addition, participants in the control condition experience increase in their RR intervals when they answered travel items questions as compared to personal information questions.

**FIGURE 9:**
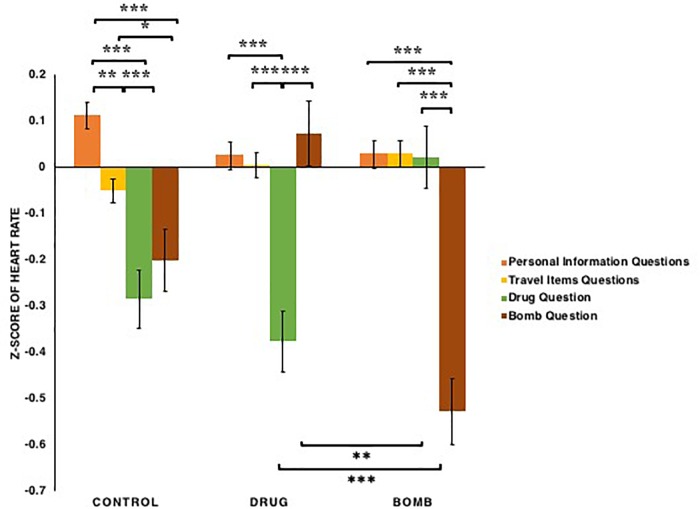
The *z*-scores of heart rates among participants in the bomb, control, and drug conditions across the four types of questions: basic personal information, carrying common travel items, carrying drugs, carrying a bomb. Note that ^∗^*p* < 0.05, ^∗∗^*p* < 0.01, ^∗∗∗^*p* < 0.001. The error bars SEs.

For the heart rate (see Figure 9), the types of questions had a significant main effect: *F*(3,363) = 20.649, *p* < 0.001, ${\text{η}}_{\text{p}}^{2}$ = 0.146, as did the conditions × types of questions interaction: *F*(6,363) = 15.913, *p* < 0.001, ${\text{η}}_{\text{p}}^{2}$ = 0.208. This interaction appeared to reflect the fact that for the participants in both drug [*t*s(38) < -4.91, *p*s < 0.001, *Bonferroni correction*] and bomb conditions [*t*s(43) < -6.36, *p*s < 0.001, *Bonferroni correction*], their heart rates when answering questions that matched their crimes were lower than when answering other questions, whereas for the participants in the control condition, heart rates when answering illegal items questions were lower than when answering other questions: *t*s(40) < -2.10, *p*s < 0.05. However, the heart rates of participants in both drug and bomb conditions when answering questions that mismatched their crimes were higher than in other conditions: *t*s(121) > 2.76, *p*s < 0.01. Additionally, for the participants in the control condition, heart rates when answering the travel items questions were lower than when answering personal information questions: *t*(40) = -2.89, *p* = 0.005. Thus, these results provide evidence that all participants experience decrease in their heart rates when answering illegal items questions, especially for those in drug and bomb conditions when answering questions that matched their crimes. In addition, participants in the control condition experienced decrease in heart rates when answering travel items questions as compared to personal information questions.

**FIGURE 10:**
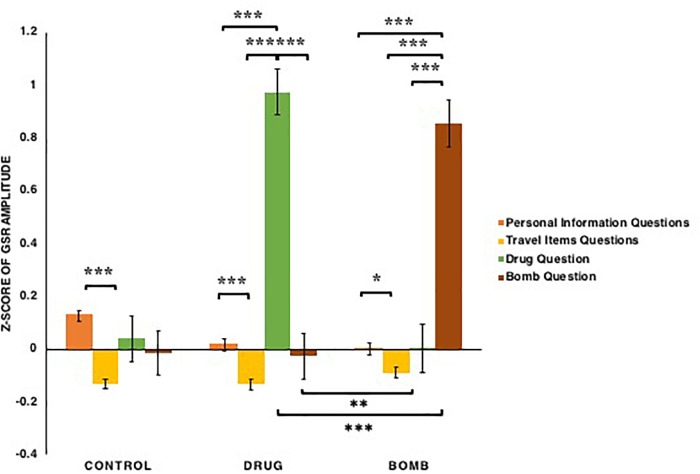
The *z*-scores of the peak-to-peak amplitude of GSR among participants in the bomb, control and drug conditions across the types of questions: basic personal information, carrying common travel items, carrying drugs, carrying a bomb. Note that ^∗^*p* < 0.05, ^∗∗^*p* < 0.01, ^∗∗∗^*p* < 0.001. The error bars SEs.

For peak-to-peak amplitude of GSR (see Figure 10), both conditions [*F*(2,121) = 16.779, *p* < 0.001, ${\text{η}}_{\text{p}}^{2}$ = 0.217] and types of questions [*F*(3,363) = 30.215, *p* < 0.001, ${\text{η}}_{\text{p}}^{2}$ = 0.200] had significant main effects, as did the conditions × types of questions interaction: *F*(6,363) = 39.140, *p* < 0.001, ${\text{η}}_{\text{p}}^{2}$ = 0.393. This interaction appeared to reflect the fact that for the participants in both drug [*t*s(38) > 7.76, *p*s < 0.001, *Bonferroni correction*] and bomb conditions [*t*s(43) > 7.03, *p*s < 0.001, *Bonferroni correction*], the galvanic skin response amplitudes when answering questions that matched their crimes were detected to be greater than when answering other questions, whereas in the control condition, galvanic skin response amplitudes when answering illegal items questions did not differ significantly from one another; furthermore, galvanic skin response amplitudes of participants in both the drug and bomb conditions when answering questions that matched their crimes were greater than in other conditions: *t*s(121) > 7.22, *p*s < 0.01. Additionally, all participants’ galvanic skin response amplitudes when answering personal information questions were detected as greater than when answering travel items questions in the control condition [*t*(40) = 6.29, *p* < 0.001, *Bonferroni correction*], in the drug condition [*t*(38) = 3.61, *p* < 0.001, *Bonferroni correction*], and in the bomb condition [*t*(43) = 2.26, *p* = 0.026]. Thus, these results provide evidence that in both drug and bomb conditions, participants’ galvanic skin response amplitudes increased when they answered questions that matched their crimes, and in all conditions, participants’ galvanic skin response amplitudes increased when they answered personal information questions compared to the travel items questions.

##### Group classifications

Discriminant Analyses (DA) were conducted to predict the classification of participants between every two of three conditions (control, drug, and bomb) by building functions with each one of three and all physiological indices across types of questions, respectively. The DA results (see Table 3) revealed significant differences between every two of three conditions of the canonical discriminant functions, which could be used in the analysis: between the control and drug conditions, Wilk’s *λ*s < 0.796, *p*s < 0.01; between the control and bomb conditions, Wilk’s λs < 0.686, *p*s < 0.001; and between the drug and bomb conditions, Wilk’s λs < 0.567, *p* < 0.001. The classification results (see Table 3) using all indices revealed that for classification of participants as either drug traffickers or common travelers (participants in the drug/control conditions), we achieved a Hit rate of 82.1% and False Alarm rate of 17.9% for drug traffickers, as well as a Hit rate of 95.1% and a False Alarm rate of 4.9% for common travelers. For classification of participants as either terrorists or common travelers (participants in the bomb/control conditions), we achieved a Hit rate of 93.2% and a False Alarm rate of 6.8% for terrorists, and a Hit rate of 95.1% and a False Alarm rate of 4.9% for common travelers. Finally, for classification of participants as either drug traffickers or terrorists (participants in the drug/bomb conditions), we achieved a Hit rate of 92.3% and a False Alarm rate of 7.7% for drug traffickers, and a Hit rate of 90.9% and a False Alarm rate of 9.1% for terrorists. The corresponding standardized canonical discriminant function coefficients between every two of three conditions are presented in Table 4. The physiological indices that contributed most, with largest coefficients, to the classification of drug traffickers/common travelers were RR intervals and heart rates when answering travel items questions, as well as RR intervals when answering drug and personal information questions. For classification of terrorists/common travelers, the physiological indices that contributed most were RR intervals and heart rates when answering personal information questions, as well as RR intervals when answering bomb and travel items questions. Finally, for classification of drug traffickers/terrorists, the physiological indices that contributed most were RR intervals and heart rates when answering personal information and travel items questions.

**Table 3:** Hit rates (i.e., correct classification rates) of participants in every two of three conditions, using one of three and all physiological indices in Experiment 2.

Condition 1 vs. Condition 2	Indices	Wilk’s λ	*p*	Hit rate of condition 1	Hit rate of condition 2
Drug condition vs. Control condition	RR interval	0.735	<0.001	79.5%	75.6%
	Heart rate	0.796	0.002	69.2%	65.9%
	GSR amplitude	0.619	<0.001	71.8%	87.8%
	All	0.492	<0.001	82.1%	95.1%
Bomb condition vs. Control condition	RR interval	0.525	<0.001	79.5%	82.9%
	Heart rate	0.686	<0.001	70.5%	70.7%
	GSR amplitude	0.615	<0.001	68.2%	87.8%
	All	0.355	<0.001	93.2%	95.1%
Drug condition vs. Bomb condition	RR interval	0.462	<0.001	84.6%	90.0%
	Heart rate	0.567	<0.001	82.1%	86.4%
	GSR amplitude	0.455	<0.001	82.1%	93.2%
	All	0.416	<0.001	92.3%	90.9%

**Table 4:** Standardized canonical discriminant function coefficients in Experiment 2.

	Function		
Comparison between conditions	Drug condition vs. Control condition	Bomb condition vs. Control condition	Drug condition vs. Bomb condition
RR Interval of Personal Information Questions	1.96	2.997	8.602
RR Interval of Travel Items Questions	0.848	1.694	7.286
RR Interval of Drug Question	0.974	-0.061	0.891
RR Interval of Bomb Question	0.243	1.055	2.671
Heart Rate of Personal Information Questions	-0.514	1.553	9.854
Heart Rate of Travel Items Questions	-1.221	0.705	8.741
Heart Rate of Drug Question	0.125	0.158	2.011
Heart Rate of Bomb Question	-	-	1.928
GSR Amplitude of Personal Information Questions	-0.795	-	0.037
GSR Amplitude of Travel Items Questions	-0.701	0.394	-
GSR Amplitude of Drug Question	0.355	0.042	-
GSR Amplitude of Bomb Question	-0.155	0.747	-

*The null value meant that the corresponding index failed the tolerance test.*

Thus, Experiment 2 revealed that guilty participants (drug and bomb conditions) experienced physiological changes consistent with those indicative of the OR. Specifically, classification based on Discriminant Analyses using RR interval, heart rate, peak-to-peak amplitude of GSR, and all three combined have revealed decelerated heart rates and increased peak-to-peak amplitude of GSR in drug and bomb condition participants when responding to the question that matched their crime. Whereas, control condition participants showed accelerated heart rates and increased peak-to-peak amplitude of GSR in response to personal information questions. In addition, accurate classification of participants between every two of three conditions (control, drug, and bomb) were: drug versus control conditions, with an 82.1% accuracy for drug traffickers and a 95.1% accuracy for common travelers; bomb versus control conditions, with a 93.2% accuracy for terrorists and a 95.1% accuracy for common travelers; drug versus bomb conditions, with a 92.3% accuracy for drug traffickers and a 90.9% accuracy for terrorists. Thus, our Modified-CQT, when used in conjunction with the polygraph test, is an affective paradigm for determining different types of guilt as well as innocence.

## General Discussion

In two experiments, we showed that Modified-CQT and the polygraph test can effectively differentiate between common travelers, drug traffickers, and terrorists based on physiological responses to critical questions significant to the crime. Specifically, we found decelerations in heart rate and increases in peak-to-peak amplitude of GSR in response to questions regarding a specific crime to be consistent with trends of an Orienting Response (OR) and thus indicative of guilt in committing the same crime. Further, our Discriminant Analyses (DA) yielded high accuracy rates for identifying innocent, drug carrying, and bomb carrying participants, with classification based on each physiological index yielding accuracy above chance level. These results are especially significant with consideration to the increasing need for transportation authorities to successfully detect intentions of drug trafficking or terrorism before they are acted upon.

Additionally, the results of Experiment 2 directly addressed the concern from Experiment 1 that Modified-CQT and the polygraph may incorrectly identify someone as innocent if they committed a different crime. With addition of a second guilty condition to carry a fake bomb, Experiment 2 showed the Modified-CQT and polygraph to be capable of differentiation between multiple types of crimes, which commonly occurs in real life. While the addition of the second guilty condition also required the addition of a question regarding possession of a bomb, it does not diminish the significance of the results. Currently, the two most pervasive and threatening crimes to occur at transportation hubs are drug trafficking and terrorism, thus our results point to the applicability of the paradigm and the polygraph for use by transportation authorities. Of course, should there become a need to detect other crimes, authorities need only include questions regarding the specific crimes to assess physiological responses to the crime and detect criminals.

Of course, while the present study’s results highlight the potential value of using the Modified-CQT in conjunction with the polygraph test for conducting airport security investigations into those suspect of carrying illegal items, several limitations should be acknowledged. First, both experiments included a rather small sample of participants, with few males (Experiment 1: 78 participants with 10 males; Experiment 2: 164 participants with 24 males). With such a small sample and an unbalanced gender distribution, it is difficult to determine whether the results can be generalized to a broader population. There is a need to assess the effectiveness of the Modified-CQT with more people, particularly more males.

Second, participants in the drug and bomb conditions only encountered one critical question in which they need to lie regarding possession of either drug or bomb. This may not be sufficient for interpretation of deceptive response. Future research should consider the addition of more questions regarding the illegal item for which participants are asked to lie about.

Third, both experiments only assessed three physiological indices and all three combined. However, there are other indices which may provide further details to help detect the possession of illegal items, including features of Heart Rate Variability (e.g., RMSSD, SDNN, and pNN50) and GSR (peak amplitude, standard deviation, mean, and variation of wave), and respiratory signals from the GSR and ECG data. Future research may wish to analyze more physiological indices to see it they would yield higher accuracy for lie detection.

Fourth, the present study only utilized ECG and GSR to observe for physiological changes related to lying. There are many other instruments that have also been shown to be effective at lie detection. Future research should consider assessments of further autonomic correlates, such as respiration and blood pressure (Gamer, 2011), or neural correlates using fMRI and fNIRS (Hu X.S. et al., 2012; Farah et al., 2014; Bhutta et al., 2015; Hong and Santosa, 2016; Hong and Khan, 2017; Hong et al., 2017, 2018) to increase the modality of measurements to further improve the rate of accuracy for identifying liars.

Fifth, while Discriminant Analyses (DA) is still used as a classifier within lie detection research (Farahani and Moradi, 2013), other classifiers, such as Support Vector Machine (SVM) have been shown to be more stable and accurate (Khan and Hong, 2015; Kaushal and Chuahan, 2016). Future research should consider conduct predictions of classification using SVM or other machine learning methods to see if higher classification accuracy can be achieved.

Sixth, despite our efforts to create a scenario as naturalistic as possible, the current experimental design still lacked the high stakes component crucial to laboratory simulated lie detection research. Future research should consider having Experimenter 1 provide monetary incentive for those in the drug and bomb condition to successfully pass the security screening. Such a design may give participants more motivation to lie well and it would be similar to real life in that a smuggler would be paid once they have successfully delivered drugs. Ultimately, the addition of a high stake component may have increased the authenticity of the situation.

Seventh, while Modified-CQT is easy to administer at train stations and airports alike, the polygraph may prove to be difficult because it requires (1) the attachment of electrodes and (2) the expertise of trained professionals to analyze the ECG and GSR signals and ascertain the physiological responses to questions of Modified-CQT. This directly challenges the claim that Modified-CQT and polygraph can make the customs process more efficient because though asking travelers questions will be faster than searching through their suitcases, the process of attaching electrodes can to tedious, inconvenient, and uncomfortable for travelers. In addition, the need for experts to analyze physiological signals is more expensive than transportation authorities randomly searching suitcases and/or bodies.

These difficulties in administering the polygraph has also been commented on by Pavlidis et al. (2002), motivating them to use a thermal imaging technique to detect lies remotely. In fact, developments in facial thermophysiology have shown facial perspiration (an indicator of stress) as obtained through thermal images to be successful in detecting lies (Dcosta et al., 2015). Recent research has also led to development of a novel contactless technology called, Transdermal Optical Imaging, to assess and monitor psychophysiological changes. The technology only requires the use of facial videos captured by conventional digital cameras, such as those on surveillance cameras, to determine heart rate, stress level, and emotions (Lee and Zheng, 2016; Wei et al., 2018). Future research should evaluate the use of such contactless lie detection methods in conjunction with Modified-CQT in real-life settings such as at airport to assess their effectiveness in detecting crimes.

## Conclusion

The present study examined the effectiveness of Modified-CQT and the polygraph test for detecting whether participants in a mock customs scenario are carrying illegal items, specifically fake drugs or bomb. We found that physiological responses, specifically changes in RR interval, heart rate, and peak-to-peak amplitude of GSR, to questions of Modified-CQT effectively indicated participants as innocent travelers or guilty criminals, including whether they were smugglers or terrorists. In addition, our Discriminant Analyses yielded high rates of accuracy in classifying participants as innocents, smugglers, and terrorists. Thus, the present findings reveal Modified-CQT and the polygraph to be valid methods for lie detection, which can be further developed to allow more efficiency for use in real life settings such as train stations and airports where transportation authorities are in need of easy and valid ways to identify criminals.

## Author Contributions

RY, GF, and KL: experimental design and data collection. RY, SW, AH, NG, HH, GF, and KL: experimental data analysis. RY, SW, GF, and KL: manuscript writing.

## Conflict of Interest Statement

The authors declare that the research was conducted in the absence of any commercial or financial relationships that could be construed as a potential conflict of interest.

## Appendix 1: Questions

### Personal Information Questions

These questions were formed based on the questionnaire (see Appendix 2) that each participant filled in. Participants were instructed to answer all questions truthfully: PQ1, PQ3, PQ7, and PQ9 for positive responses, PQ2, PQ4, PQ8, and PQ10 for negative responses, PQ5 and PQ6 for actual responses.

PQ1/PQ2: Do you major in ×× (major)?
PQ3/PQ4: Is your hometown in ×× (province)?
PQ5: Do you have sibling?
PQ6: Are you an only child?
PQ7/PQ8: Have you ever been to ×× (city)?
PQ9/PQ10: Have you ever been to the University of ×× (university)?

### Context-Relevant Questions

These questions were formed based on the existence/non-existence of items in the suitcase. Participants were instructed to answer these questions truthfully in the control condition, while in the drug condition except CQ1 and in the bomb condition except CQ2. (There was no CQ2 in Experiment 1).

CQ1: Did you pack any drugs in your suitcase?
CQ2: Did you pack a bomb in your suitcase?
CQ3: Did you pack clothes in your suitcase?
CQ4: Did you pack a knapsack in your suitcase?
CQ5: Did you pack books in your suitcase?
CQ6: Did you pack an umbrella in your suitcase?
CQ7: Did you pack toiletry kits in your suitcase?
CQ8: Did you pack a belt in your suitcase?
CQ9: Did you pack sandals in your suitcase?
CQ10: Did you pack socks in your suitcase?
CQ11: Did you pack sunglasses in your suitcase?
CQ12: Did you pack a hat in your suitcase?

## Appendix 2: Questionnaire

### Instruction

This is a short questionnaire to provide us with some personal information about you. Please answer these questions honestly because your answers will be used to form questions for a polygraph test. None of the information you provide here will be shown to anyone else or linked back to you. If you do not feel comfortable answering a question, you may skip it. Please submit the questionnaire to the researcher once you are done.

Please write down your major:

_________________________________________________

Please write down your hometown:

_________________________________________________

Do you have siblings?

_________________________________________________

Please list at least one city that you have been to:

_________________________________________________

Please list at least one city that you have not yet been to:

_________________________________________________

Please list at least one university that you have been to:

_________________________________________________

Please list at least one university that you have not yet been to:
